# Pediatric patients with RASopathy-associated hypertrophic cardiomyopathy: the multifaceted consequences of *PTPN11* mutations

**DOI:** 10.1186/s13023-019-1151-0

**Published:** 2019-07-05

**Authors:** Giulio Calcagni, Maria Cristina Digilio, Bruno Marino, Marco Tartaglia

**Affiliations:** 1 Cardiology Unit, Department of Pediatric Cardiology and Cardiac Surgery, Bambino Gesù Children’s Hospital and Research Institute, Viale di San Paolo 15, 00146 Rome, Italy; 2 Genetics and Rare Diseases Research Division, Bambino Gesù Children’s Hospital and Research Institute, Viale di San Paolo 15, 00146 Rome, Italy; 3 Pediatric Cardiology, Department of Pediatrics, Sapienza University, Viale Regina Elena 324, 00161 Rome, Italy

**Keywords:** PI3K-AKT-mTOR, MAPK, Hypertrophic cardiomyopathy, RASopathy

## Abstract

The concomitant occurrence of hypertrophic cardiomyopathy and congenital heart defect in patients with RASopathies has previously been reported as associated to a worse clinical outcome, particularly closed to cardiac surgery. Different mechanisms of disease have been demonstrated to be associated with the two classes of *PTPN11* mutations underlying Noonan syndrome and Noonan syndrome with multiple lentigines (also known as LEOPARD syndrome). Although differential diagnosis between these two syndromes could be difficult, particularly in the first age of life, we underline the relevance in discriminating these two disorders in terms of affected signaling pathway to allow an effective targeted pharmacological treatment.

Dear Editor,

We read with interest a recent research by Chen and colleagues [1] reporting on the clinical and mutation profiles of pediatric patients with RASopathy-associated hypertrophic cardiomyopathy (HCM). In this manuscript, the authors described the mutation spectrum causally linked to Noonan syndrome (NS) (MIM PS163950) and clinically related disorders, and the associated clinical outcome, based on a pediatric cohort of 47 affected subjects. An exhaustive genotype-phenotype correlation was reported. Particularly, the data allowed the authors to emphasize further the relevant contribution of the concomitant occurrence of congenital cardiac defects (CHDs) and left ventricular outflow tract obstruction (LVOTO) to the worse outcome of these patients. The authors also reported that patients with a diagnosis of NS based on clinical criteria and carrying two specific missense mutations in *PTPN11* (c1417A > C, p.Q506P; c1528C > G, p.Q510E) showed an early-onset presentation of HCM and a worse clinical outcome. Indeed, out of this subgroup of eleven patients, three of them (p.Q506P, *N* = 1; p.Q510E, *N* = 2) died within 6 months from birth.

We agree with Chen and colleagues that the co-occurrence of HCM and CHDs is generally associated to a worse outcome in patients with RASopathies. Indeed, these patients may show a rapid progression of HCM and this can lead to early cardiac failure. These data are also in line with other recently published studies [2–4]. In particular, our experience confirms that a worse clinical outcome is strictly closed to complex cardiac surgery [2]. However, we would point out that the biochemical/functional behavior of *PTPN11* mutations at codons 510 (including c.1528C > G) and 506 is drastically different from what observed for NS-causing *PTPN11* mutations. Specifically, the former cause defective protein phosphatase activity and differentially affect intracellular signaling [5–7]. Consistent with their distinctive consequences on SHP2 function and signal transduction, these mutations do not cause NS but underlie Noonan syndrome with multiple lentigines (NSML), previously known as LEOPARD syndrome (MIM PS151100), a disorder similar but distinct from NS. NSML-associated *PTPN11* mutations cluster within or close to the active site of the phosphatase, which explains their dramatic impact on phosphatase activity when compared with wild-type SHP2 and NS-causing *PTPN11* mutations [5, 8, 9]. It should be noted that the presence of cutaneous manifestations as café-au-lait spots and multiple lentigines, which represent a distinctive feature of NSML, develop with age and do not generally occur in infancy. Based on this consideration, the absence of lentigines during infancy should not be used to exclude a diagnosis of NSML. HCM is an additional common complication of NSML. Differently from what reported by Chen and colleagues [1], it is now well-established, and also in line with previous reports [10], that c.1528C > G (p.Q510E) in *PTPN11* is strictly associated to NSML. Review of published cases carrying the c.1528C > G missense change documents that most of the patients have a clinical diagnosis of NSML syndrome (Table 1) [11–14]. Notably, two among the three reported patients classified as having NS presented with HCM associated with deafness, a clinical feature characteristic for NSML (Table 1) [15–17]. In regard to c.1529A > C and c. 1530 G > C, also involving codon 510 of *PTPN11*, it should be noted that all of them have been reported in patients diagnosed as having NSML syndrome (Table 1) [18–21].

**Table 1:** Literature reports of patients carrying mutation at codon 510 of *PTPN11* gene

Mutation	Age at observation	Reported clinical diagnosis	Clinical features specific for NS-ML	Reference
c.1529A > C; p.Q510P	Adult	NS-ML	Lentigines	Keren et al., 2004
c.1529A > C; p.Q510P	12 years	NS-ML	Deafness Lentigines Café-au-lait spots Heart defect and ECG anomalies	Keren et al., 2004
c.1529A > C; p.Q510P	25 years	NS-ML	Deafness Lentigines Café-au-lait spots PVS	Keren et al., 2004
c.1528C > G; p.Q510E	14 months	NS	HCM Deafness	Takahashi et al., 2005
c.1529A > C; p.Q510P	Adult	NS-ML	Lentigines	Kalidas et al., 2005
c.1529A > C; p.Q510P	Adult	NS-ML	Deafness Lentigines	Kalidas et al., 2005
c.1529A > C; p.Q510P	1 year	NS-ML	PVS, ASD, ECG anomalies	Kalidas et al., 2005
c.1528C > G; p.Q510E	2 years	NS-ML	HCM Café-au lait spots Lentigines	Digilio et al., 2006
c.1528C > G; p.Q510E	2 years	NS-ML	HCM Café-au-lait spots	Digilio et al., 2006
c.1528C > G; p.Q510E	2 months	NS	HCM	Faienza et al., 2009
c.1528C > G; p.Q510E	37 years	NS-ML	HCM Lentigines Deafness	Lehmann et al., 2009
c.1528C > G; p.Q510E	5 years	NS	HCM, PVS Deafness	Derbent et al., 2010
c.1529A > C; p.Q510P	4 years	NS or NS-ML	Café-au-lait spot	Brasil et al., 2010
c.1528C > G; p.Q510E	infant	NS-ML	HCM	Ganigara et al., 2011
c. 1530 G > C; p.Q510H	38 years	NS-ML	HCM, PVS, ASD Lentigines Cafe-au-lait spots	Wakabayashi et al., 2011
c.1528C > G; p.Q510E	20 months	NS-ML	HCM Deafness	Hahn et al., 2015

*ASD* atrial septal defect, *ECG* electrocardiogram, *HCM* hypertrophic cardiomyopathy, *ML* multiple lentigines, *NS* Noonan syndrome, *PVS* pulmonary valve stenosis

While NS and NSML are genetic conditions with overlapping features, and clinicians experienced with these syndromes are aware of the difficulty in discriminating between these two disorders particularly in the first years of age [11, 22], we firmly believe that it is important to properly discriminate NSML from NS to allow a more effective patient management and a future personalized pharmacological treatment of the evolutive complications in these patients. Indeed, it is important to consider that different mechanisms of disease have been demonstrated to be associated with the two classes of *PTPN11* mutations underlying NS and NSML. On one hand, we have hyperactive mutants promoting upregulation of MAPK signaling (NS-causing mutations), which can effectively be controlled by the use of inhibitors of signal transducers functioning in this cascade (e.g., MEK1 inhibitors) [23, 24]. On the other hand, we have hypomorphic mutants that result in enhanced signal flow through the PI3K-AKT-MTOR pathway (NSML-causing mutations). In this case, inhibitors targeting this specific cascade (e.g., AKT inhibitors, rapamycin analogs) are required to counterbalance and treat evolutive complications of NSML, including HCM [14, 25, 26]. On this argument, Wang et al. [26] reported an in vivo study suggesting that the AKT inhibitor ARQ 092 may be a promising novel therapy for treatment of hypertrophy in NSML patients. Additionally, Hahn and colleagues [14] reported the effects of treatment with a rapamycin analog in an infant with NSML and severe HCM.

In summary, we should avoid the misleading causative association between NSML-causing *PTPN11* mutations and NS, based on the diverse impact of these mutations on SHP2 function and intracellular signaling dysregulation, and their consequent significance in terms of patient management and future personalized therapeutic options.

## Data Availability

Not applicable.

## Abbreviations

CHDs: Congenital cardiac defects
HCM: Hypertrophic cardiomyopathy
LVOTO: Left ventricular outflow tract obstruction
NS: Noonan syndrome
NSML: Noonan Syndrome with Multiple Lentigines
